# Bibliography

**Published:** 1901-03-15

**Authors:** 


### BIBLIOGRAPHY.

Principles and Practice of Filling Teeth, by C. N. Johnson, Prof. of Operative Dentistry in the Chicago Dental College.—It is an octavo volume of nearly three hundred pages, neatly printed and bound in cloth. It is published by the S. S. White Dental Manufacturing Co., Philadelphia.

This work is not a complete treatise on operative dentistry, neither is it a text book on this subject; and yet it embodies some of the essential elements of both. It seems to be a kind of "heart to heart" talk on modern operative proceedings, largely from the technical standpoint. At the same time it systematically considers sequentially the various operations demanded by the progress of dental caries. While it is probable that it may not become popular as a teachers' hand-book or students' guide, it has features which will make of it an invaluable reference work. It is particularly adapted to the practitioner, and ought to be in the hands of every dentist, for it contains much in the way of valuable suggestion which will tend to eliminate errors and perfect judgment in the development of systematic technique. There is very little to criticise in the work, and there is so much that should be commended, that we can not take time to condemn when there is so much to support.

The first chapter is devoted to deposits on the teeth and their removal, and is well treated. The next chapter, on dental caries, is largely an etiological statement, which is perhaps the only digression from what might be considered the avowed purpose of the work. The third chapter takes up the purely operative work in an all too limited discussion of methods of examining the teeth for caries. This chapter might very appropriately be enlarged by adding the principles underlying correct diagnosis and a statement as to its use and importance. The next chapter is devoted to a consideration of the "methods used in excluding moisture." It is well written and complete. Chapter V is probably the most important in the entire work. Nearly seventy-five pages are devoted to the "Classification and Preparation of Cavities." It is full of helpful suggestions, and yet some things are emphasized to which many good operators will take exception. This chapter was published in the *Dental Cosmos* in 1898 and 1899, and needs no further comment here. Two chapters are given up to a consideration of filling materials. Gold, amalgam, tin, cements and gutta-percha are all briefly but satisfactorily treated. The chapter on introduction and finishing of gold fillings is a long one and carefully considers the difficulties encountered in filling cavities in all possible situations. We do not agree with the author in much of this chapter, but it is largely because we were trained to manipulate a different form of gold, and our methods of securing similar results differ. Here possibly many practitioners will not agree with the author, but a careful study of his methods will possibly lead to a change of habitual practice for the better. For instance, the author states it as a principle that the first piece of gold placed in a cavity should serve as a cushion upon which the subsequently added gold should be built. To many this idea is fundamentally at variance with correct mechanical principles. To those who have depended upon the firm anchorage of the starting point or pit this may suggest insecurity, but much depends on the preparation of the cavity as well as the character of subsequent manipulations.

Further chapters are devoted to the manipulation of amalgam, cement and gutta-percha as filling materials. There are also chapters briefly describing the insertion of *inlays*, *pulp capping*, *destruction of pulps*, and on the operative management of pulpless teeth. The subject of opening, disinfecting and management of pulp canals is not as comprehensively treated as its importance demands. The last ten pages are devoted to the management of children's teeth. This important subject is also greatly overlooked, not only here, but in every other work on this subject. The importance of preserving intact the deciduous teeth until the proper time for their loss is not well understood, in spite of the fact that much has been written on this subject.

In this work Dr. Johnson has made us debtor for the thoroughly candid manner in which he has expressed his convictions and practices. The book is entertainingly written and can be read with profit as well as pleasure by any one. We think it will appeal most strongly to the practitioner.

